# A higher-protein nut-based snack product suppresses glycaemia and decreases glycaemic response to co-ingested carbohydrate in an overweight prediabetic Asian Chinese cohort: the Tū Ora postprandial RCT

**DOI:** 10.1017/jns.2021.20

**Published:** 2021-04-23

**Authors:** Louise W. Lu, Marta P. Silvestre, Ivana R. Sequeira, Lindsay D. Plank, Meika Foster, Nikki Middleditch, Alejandra Acevedo-Fani, Kieren G. Hollingsworth, Sally D. Poppitt

**Affiliations:** 1 Human Nutrition Unit, School of Biological Sciences, University of Auckland, Auckland, New Zealand; 2 High-Value Nutrition National Science Challenge, Auckland, New Zealand; 3 Department of Surgery, University of Auckland, Auckland, New Zealand; 4 Edible Research Ltd, Christchurch, New Zealand; 5 Department of Medicine, University of Otago, Dunedin, New Zealand; 6 Riddet Institute, Massey University, Palmerston North, New Zealand; 7 Newcastle Magnetic Resonance Centre, Institute of Cellular Medicine, Newcastle University, Newcastle upon Tyne, UK; 8 Riddet Centre of Research Excellence (CoRE) for Food and Nutrition, Palmerston North, New Zealand; 9 Department of Medicine, University of Auckland, Auckland, New Zealand

**Keywords:** Dried fruits, MRI, Nuts, Postprandial glycaemia, Prediabetes, AUC, area under the curve, BF, body fat, BMI, body mass index, CHO, carbohydrate, DXA, dual-energy X-ray absorptiometry, GI, glycaemic index, iAUC, incremental area under the curve, MRI, magnetic resonance imaging, MRS, magnetic resonance spectroscopy, SAT, subcutaneous adipose tissue, T2D, type 2 diabetes, VAS, visual analogue scales, VAT, visceral adipose tissue, WB, white bread

## Abstract

Nut-based products may aid low-glycaemic dietary strategies that are important for diabetes prevention in populations at increased risk of dysglycaemia, such as Asian Chinese. This randomised cross-over trial assessed the postprandial glycaemic response (0–120 min) of a higher-protein nut-based (HP-NB) snack formulation, in bar format (1009 kJ, Nutrient Profiling Score, NPS, −2), when compared with an iso-energetic higher-carbohydrate (CHO) cereal-based bar (HC-CB, 985 kJ, NPS +3). It also assessed the ability to suppress glucose response to a typical CHO-rich food (white bread, WB), when co-ingested. Ten overweight prediabetic Chinese adults (mean, sd: age 47⋅9, 15⋅7 years; BMI 25⋅5, 1⋅6 kg/m^2^), with total body fat plus ectopic pancreas and liver fat quantified using dual-energy X-ray absorptiometry and magnetic resonance imaging and spectroscopy, received the five meal treatments in random order: HP-NB, HC-CB, HP-NB + WB (50 g available CHO), HC-CB + WB and WB only. Compared with HC-CB, HP-NB induced a significantly lower 30–120 min glucose response (*P* < 0⋅05), with an approximately 10-fold lower incremental area under the glucose curve (iAUC_0–120_; *P* < 0⋅001). HP-NB also attenuated glucose response by approximately 25 % when co-ingested with WB (*P* < 0⋅05). Half of the cohort had elevated pancreas and/or liver fat, with 13–21 % greater suppression of iAUC_0–120_ glucose in the low *v.* high organ fat subgroups across all five treatments. A nut-based snack product may be a healthier alternative to an energy equivalent cereal-based product with evidence of both a lower postprandial glycaemic response and modulation of CHO-induced hyperglycaemia even in high-risk, overweight, pre-diabetic adults.

The prevalence of type 2 diabetes (T2D) has dramatically increased from 110 million people in 1994 to 463 million in 2019, with almost 10 % of the global adult population now affected^(1)^. T2D and adverse metabolic health have become critical healthcare and economic problems for Asia, with China in particular rapidly shouldering the most substantial global burden^(2)^, where T2D prevalence has already reached 100 million^(3)^. When Asian Chinese consumers are exposed to Westernised food environments and lifestyle, they are particularly susceptible to the development of a myriad of problems, including weight gain, poor metabolic health, T2D and cardiovascular disease (CVD). Adverse abdominal and ectopic lipid storage is purported, at least in part, to underpin this increased susceptibility^(4)^. Long-term exposure to repeated acute postprandial hyperglycaemia has long been established as a risk factor for multiple metabolic disorders^(5)^, with evidence of adverse glycaemic excursions in Asian cohorts^(6–8)^.

Lowering the glycaemic index (GI) or glycaemic load (GL) of a meal can positively impact the postprandial blood glucose excursions in Asian Chinese and, in turn, potentially reduce the risk of T2D and CVD^(9)^. Prior epidemiological^(10)^ and randomised controlled trials (RCTs) have linked the intake of tree nuts^(10–14)^ and peanuts^(12,15–17)^ to a decreased risk of T2D and CVD^(13)^. Nuts are a good source of high-quality plant protein, and mono- (MUFA) and polyunsaturated fatty acids (PUFA)^(18)^, all of which are proposed to improve blood lipid profile, decrease insulin resistance (IR), decrease inflammation and oxidative stress and modulate endothelial function^(10)^, as well as potentially promoting satiety^(17,19)^. Of particular interest are prior studies that have shown that tree nuts, including almonds, consumed at different doses can suppress postprandial glycaemia induced by high carbohydrate (CHO) foods^(20,21)^. An acute study reported that the addition of pistachios to a white bread (WB) meal resulted in a dose-dependent decrease in the postprandial glycaemic response in ten healthy participants^(20)^. Another dose-response study in healthy participants also confirmed that the consumption of almonds with WB resulted in a decrease in postprandial glycaemia, with approximately 1⋅0 mM reduction in peak circulating glucose, despite the increase in total available CHO content^(21)^. Similarly, peanuts consumed with breakfast elicited a favourable postprandial effect on glucose response in a randomised cross-over trial of women with obesity and increased risk of T2D^(17)^. In this study, the addition of peanut butter or whole peanuts to a CHO-rich meal lowered the postprandial glucose response (iAUC_0–490 min_) by approximately 19 % and approximately 14 %, respectively, compared with a control meal without peanut butter. Co-ingestion of peanut butter or whole peanuts decreased the GI of the CHO-rich meal from approximately 61 units to approximately 56 and approximately 58 units, respectively^(17)^. In turn, dried fruits are nutritionally dense foods, rich in fibre, with minerals such as potassium and magnesium, and a wide range of phytochemicals, such as phenolic acids, flavonoids, phytoestrogens and carotenoids^(22)^, and have been proposed to contribute to lower T2D risk^(23)^. Mechanisms include an acute inhibition of postprandial α-amylase and α-glucosidase activity and longer-term changes in the gut microbiome^(23–25)^.

In our present study, we sought to evaluate the postprandial response of overweight Chinese adults, at risk of T2D, to nuts and dried fruit-based formulation provided in snack bar format and containing the recommended daily serving of 28 g of nuts^(25,26)^, both consumed alone and then co-ingested with a high CHO high GI/GL food item to investigate whether the nut-based bar has the potential to lower the glycaemic response to a meal, in comparison with a standard cereal-based bar.

## Materials and methods

This postprandial glycaemic response trial was a randomised, cross-over study conducted at the Human Nutrition Unit (HNU), University of Auckland, New Zealand, between September and December 2018. It was a sub-study of the Tū Ora Project: ‘Normalising glycaemia in overweight, pre-diabetic adults, a study of Asian Chinese adults resident in New Zealand’. The study was conducted according to the guidelines laid down in the Declaration of Helsinki, and all procedures involving human participants were approved by the National Health and Disabilities Ethics Committee (HDEC), Auckland, New Zealand (18/NTB/1/AM03). The study was prospectively registered with the Australian New Zealand Clinical Trials Registry (ACTRN12618000476235). Written informed consent was obtained from all participants. All participants received an information sheet detailing the study protocol and gave written informed consent.

### Participants

Ten overweight, pre-diabetic male and female participants were recruited into this postprandial glycaemic response trial from the greater Auckland region in New Zealand through advertising on social media platforms such as WeChat (www.wechat.com), in addition to radio and newspaper adverts. The eligibility of participants was initially assessed at pre-screen, using an online survey on Research Electronic Data Capture (REDCap, Nashville, TN, USA)^(27)^ or via telephone. The inclusion criteria were self-reported ethnic Chinese, aged between 25 and 70 years, with a body mass index (BMI) of 23–40 kg/m^2^, which was indicative of overweight and obesity in Asian cohorts^(28–30)^, a Finnish Diabetes Risk Score (FINDRISC) ≥ 9^(31)^, and fasting plasma glucose, 5⋅6–6⋅9 mM^(32^^)^. The exclusion criteria were body weight change >5 % in the previous 3 months, diagnosed with prior or current significant disease including T2D, currently taking medication affecting body weight or glucose metabolism, and tree nut/peanut or other food allergy. Participants were then screened in the clinic to confirm eligibility including the measurement of body weight and height and fasting finger-prick (FP) glucose using a handheld glucose meter (CareSens®N, iSens, New Zealand). The sample size of ten individuals was based on the ISO standards for GI assessment^(33)^. HbA_1c_ is reported for *n*=9 participants, measured within 28 d prior to the first study visit.

### Body composition assessments

#### Dual-energy X-ray absorptiometry scan

Whole-body dual-energy X-ray absorptiometry (DXA) scans were conducted prior to the start of the postprandial meal study at the Department of Surgery, University of Auckland (iDXA, software version 15, GE-Lunar, Madison, WI, USA), using a standard imaging and body positioning protocol. Whole-body scan images were analysed for total fat mass (TFM), abdominal fat mass (AbFM), total lean mass (TLM) and fat-free mass (FFM = TLM + bone mineral content, BMC). The percentage of body fat (%BF) was calculated as TFM × 100/(TFM + TLM + BMC). AbFM was determined from a region of interest defined automatically with lower horizontal boundary placed at the top of the iliac crest and height set to 20 % of the distance from this boundary to the base of the skull, with the lateral margins including the waist outline^(34)^.

#### Magnetic resonance imaging/magnetic resonance spectroscopy

Magnetic resonance imaging (MRI) was used to determine abdominal and pancreas fat, and magnetic resonance spectroscopy (MRS) was used to determine liver fat content. Fast sagittal localising abdominal images from diaphragm to pelvis were acquired using the 3D dual gradient-echo sequence (VIBE) 2-point Dixon method^(35)^ on a 3T Magnetom Skyra scanner (Siemens, Germany, VE 11A). VAT (visceral adipose tissue) and SAT (subcutaneous adipose tissue) were quantified from a single fat fraction map at the L4–L5 intervertebral disc space^(36)^ using ImageJ^(37)^. Pancreas fat was determined using the MR-opsy method^(38)^ with thresholding (1–20 %) applied to eliminate any inclusion of non-parenchymal tissue. There is no established global cut-off for the percentage of pancreas fat, in addition to which the pancreas imaging method in our study has some T1-weighting and, therefore, is expected to overestimate fat fraction^(39)^. Hence, we internally compared the percentage of pancreas fat in our cohort of women based on weighted means as reported from nine studies in a recent meta-analysis by Singh *et al.*^(40)^, and as such, ≥4⋅5 % was used to classify individuals with high pancreas fat. MRS was performed using a respiratory-gated sequence^(41)^ and liver fat was calculated using SIVIC software^(42)^ from the area under the curve (AUC) of water and fat peaks from non-water-suppressed spectra, corrected for T2-weighting according to prior literature^(43)^. Data were presented as percentage volume/volume, with a liver fat of ≥5⋅6 % identified as elevated. This cut-off has been reported previously as the upper 95 percentile that corresponds to approximately 15 % of histological liver fat in a cohort of healthy adults^(44)^.

### Study protocol

**Fig. 1.**
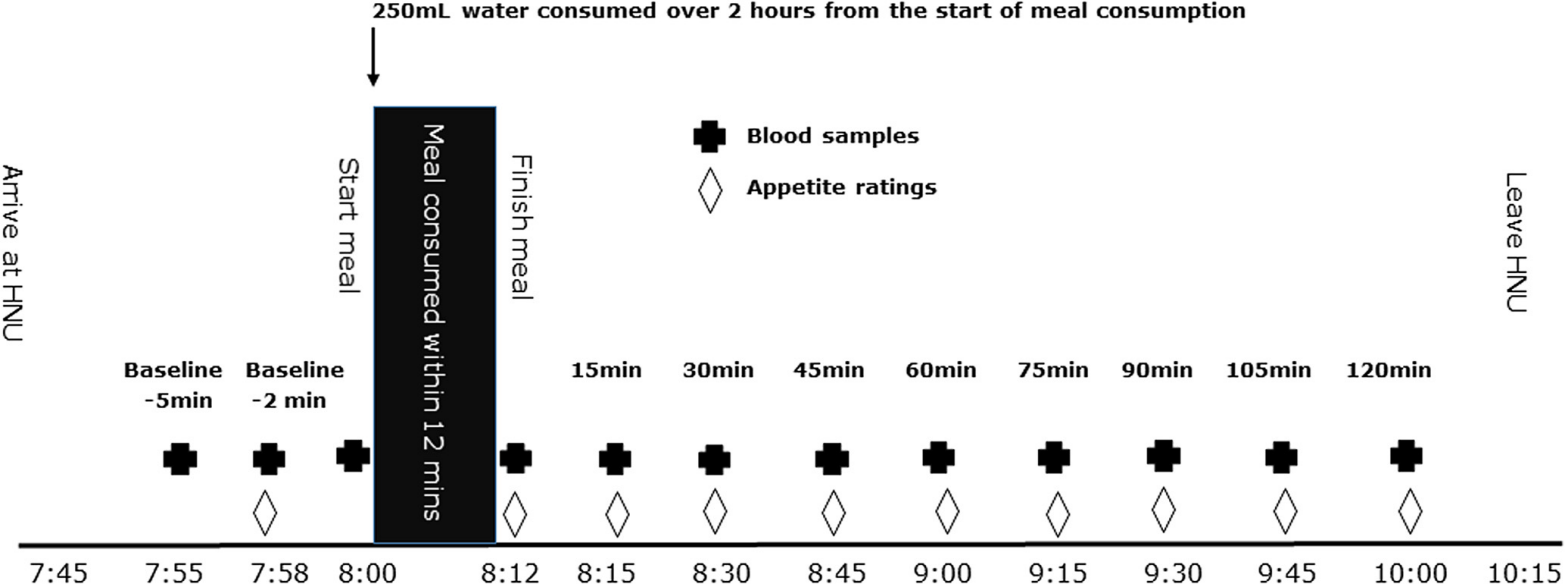
Daily protocol for postprandial blood glucose assessment.

The participants were asked to maintain their regular meal, sleep and exercise patterns throughout the duration of the study and refrain from alcoholic beverages, caffeine and strenuous exercise during the 12 h of fasting (water only) prior to and for the duration of each GI test. The five meal treatments were given to each participant on separate days in a random order determined prior to the start of the study. The randomisation list was generated on the Sealed Envelope website (Sealed Envelope Ltd. 2020, create a blocked randomisation list, https://www.Sealedenvelope.com/simple-randomiser/v1/lists). A minimum 1-d washout was required between each treatment. This was an open, un-blinded intervention. On each trial day, the participants arrived at the HNU clinic in the morning after an overnight fast and completed the 2 h postprandial test (Fig. 1). At the baseline, two FP capillary blood samples were collected for measurement of fasting blood glucose concentration (CareSens®N, iSens, New Zealand). Baseline appetite ratings were also assessed using 100 mm visual analogue scales (VAS) for hunger, fullness, satisfaction, thoughts of food (TOF), thirst, energy, relaxation and nausea^(45)^. Immediately after these baseline measures were completed, the participants began consuming the test meals at a steady pace, with the aim of completing the last bite at the 12 min-time point, under supervision. Water was provided and standardised, with all participants consuming 250 ml with each meal. VAS-palatability ratings were completed at the end of each breakfast meal. Postprandial FP blood glucose and VAS-appetite ratings were then measured over the following 2 h at 15, 30, 45, 60, 75, 90, 105 and 120 min.

To minimise variability in the primary outcome of glucose concentration, each participant was allocated a glucometer at the start of the intervention which was used for all five meal treatments. Quality control measures were performed on all glucometers at the start of each study day, using three glucose control solutions levels: Control A (low) 2⋅0–3⋅8 mM; Control B (medium) 5⋅6–8⋅4 mM; Control C (high) 10⋅2–15⋅3 mM. The CV of the four glucometers was between 3 and 10 %.

### Meal treatments

Five meal treatments were as follows: (1) higher-protein nut-based (HP-NB) test formulation, provided in snack bar format; (2) higher-CHO cereal-based bar (HC-CB), iso-energetic comparator bar; (3) HP-NB co-ingested with WB (50 g available CHO); (4) HC-CB co-ingested with WB (50 g available CHO); (5) WB only (50 g available CHO). Details of the five meals are listed in Table 1. The HP-NB (50 g per serve; 1009 kJ) ingredients were halved almonds shelled with skin, halved peanuts shelled without skin, dehydrated apple, dehydrated blueberry, chicory oligofructose (Fibrulose ® L90, Sensus, Netherlands), rolled oats, *Piper excelsum* (kawakawa) leaves, sunflower oil and soya lecithin. The HP-NB was formulated to meet healthy food guidelines and to comprise (1) recommended daily serving of nuts (>28 g/d^(26)^), (2) higher total protein, (3) higher total and unsaturated fat, (4) lower total CHO, free sugars and higher fibre, in comparison with the cereal bar. The iso-energetic HC-CB comparator (64 g/serve; 985 kJ) ingredients were rolled oats, white flour, canola oil (Davis food ingredients®, NZ), rice malt syrup (Pureharvest®, Australia) and baking soda. The comparator was formulated to comprise (1) matched energy content, (2) higher cereal content, (3) higher total CHO and free sugars (based on typical commercial cereal-based muesli bar; a mean available CHO content of thirty-two commercial ‘muesli bars’ of varying composition available from the New Zealand/Australia Foodworks dietary database, 38 g available CHO/64 g bar, range 22–46 g; Xyris Software, Melbourne, Australia), (4) lower total fat, in comparison with the nut-based bar. Both products were manufactured and provided by the High-Value Nutrition Science of Food team based at Riddet Institute using the services of Massey University FoodPILOT (Palmerston North, New Zealand) and with commercial manufacturing advice provided by the NUKU ki te Puku^TM^ cluster of Māori businesses with assistance from Griffins Food Company (Auckland, New Zealand).

**Table 1.** Energy and macronutrient composition of the five meal treatments

	HP-NB^a^			HC-CB^a^			HP-NB + WB^a^			HC-CB + WB^a^			WB^b^		
	g	kJ	Energy %	g	kJ	Energy %	g	kJ	Energy %	g	kJ	Energy %	g	kJ	Energy %
Single serve	50	1009	–	64	985	–	153	2098	–	167	2074	–	103	1089	–
Total CHO	23	270	27	40	647	64	77	1141	54	94	1518	73	54	871	80
Available CHO	12	205	20	38	635	63	62	1040	50	88	1470	71	50	835	77
Starch	7	119	12	28	475	47	54	901	43	75	1252	60	47	784	72
Sugars	5	87	9	10	160	16	8	137	7	13	210	10	3	50	5
Fibre	10	65	6	2	12	1	14	94	5	6	41	2	4	29	3
Total protein	8	130	13	4	74	7	17	289	14	13	233	11	9	159	14
Total fat	16	608	60	7	264	26	18	682	32	9	338	16	2	74	6
SFA	2	72	7	1	24	2	2⋅1	77	4	1⋅1	29	1	0⋅1	5	0⋅5
MUFA^c^	9	333	34	4	148	15	9⋅6	355	17	4⋅6	170	8	0⋅6	22	2
PUFA^c^	5	185	19	2	74	7	5⋅6	207	10	2⋅6	96	5	0⋅6	22	2
NPS^d^	−2			3			N/A			N/A			−1		

HP-NB, higher-protein nut-based bar; HC-CB, high-carbohydrate cereal-based bar; WB, white bread; CHO, carbohydrate; SFA, saturated fatty acid; MUFA, mono-unsaturated fatty acid; PUFA, polyunsaturated fatty acid; NPS, nutrition profiling score.

a Calculated, FoodWorks® (version 8, Xyris Software, Melbourne, Australia).

b WB natures fresh toast bread white (Goodman Fielder®, New Zealand).

c WB data obtained from ‘bread, white, sliced, prepacked, lower North Island’ from New Zealand Food Composition Database: New Zealand FOODfiles 2016^(46)^.

d NPS calculated using the Food Standards Australia New Zealand online NPS calculator. (https://www.foodstandards.gov.au/industry/labelling/Pages/Nutrient-Profiling-Scoring-Calculator.aspx)^(47)^. The following data were entered: (1) nutrition information, per 100 g (energy, kJ; protein, g; SFA, g; sugars, g; dietary fibre, g; sodium, mg); (2) composition of fvnl (fruit, vegetable, nuts, legumes) ingredients, non-fvnl ingredients and concentrated fruit or vegetable ingredients. For Category 2 (solid food), NPS < 4 meets the NPS criterion for Australia and New Zealand as suitable for a health claim.

### Statistical analysis

Participant characteristics were presented as mean, standard deviation (mean, sd) and efficacy endpoints as mean, standard error of the mean (mean, sem). The change in blood glucose concentration from the fasting baseline (Δ blood glucose concentration, mM) over 120 min was calculated for all individuals for all meals (Excel, Microsoft Office 365, Washington, DC, USA). The incremental AUC (iAUC) of blood glucose concentration was then calculated using the trapezoid method, including both positive and negative peaks, using Prism 8 (GraphPad Software, San Diego, CA, USA). The peak postprandial blood glucose concentration (*C*_max_, mM) for each participant at each meal was assigned using the highest postprandial blood glucose value, and the time to maximum peak (*T*_max_, min) was assigned from the glucose curve. Repeated-measures analysis of variance (RMANOVA) and Tukey's pairwise *post hoc* tests were used to examine the difference in blood glucose peaks (Δ*C*_max_, mM) and peak time (Δ*T*_max_, min) and iAUC blood glucose concentration. VAS-palatability and VAS-appetite ratings were also analysed using RMANOVA methods. Pearson correlation was used to investigate the association between pancreas and liver fat and other anthropometric and biochemical parameters. Analyses were performed as intention to treat. No participants dropped out after completion of the first study arm. DXA body composition measurement was not completed for two participants due to machine malfunction. The blood glucose sample for one-time point for one participant was not collected (study arm HP-NB + WB, *t* 15 min), with data imputed using the group mean value. The statistical significance was set at *P* < 0⋅05.

## Results

### Participant characteristics

Ten overweight, pre-diabetic Chinese participants (two males, eight females) with a mean (sd) age of 47⋅9 (15⋅7) years and BMI 25⋅5 (1⋅6) kg/m^2^ were enrolled into the study between 14 September and 8 December 2018 (Table 2). All ten participants completed the study with no adverse events. The mean (sd) FINDRISC score measured at pre-screen was 13⋅2 (3⋅2), indicating an increased risk of T2D development. At the baseline, participants had high DXA-assessed total and abdominal adiposity and a high MRI-assessed VAT:SAT ratio. Mean MRI- and MRS-assessed pancreas and liver fat were 4⋅1 (1⋅6) % and 5⋅1 (7⋅7) %, with a wide range for both. Half of the cohort (*n*=5) had elevated organ (pancreas and/or liver) fat content. Of note, one participant had significant hepatic steatosis, with 26 % liver fat. Waist circumference and MRI-assessed VAT, but not BMI or DXA-assessed total percentage of BF, were significantly associated with a high percentage of pancreas fat in the whole cohort (both, *P* < 0⋅05, data not shown). While the DXA-assessed total percentage of BF was significantly associated with the percentage of liver fat (*P* < 0⋅05), MRI-assessed VAT was borderline associated (*P=*0⋅06). Conversely, neither BMI nor waist circumference was significantly associated with the percentage of liver fat. The mean (sd) fasting FP blood glucose concentration was 6⋅0 (0⋅4) mmol/l, in the pre-diabetic range, with borderline significant correlation with the percentage of pancreas fat (*P* =0⋅09, data not shown) but not with the percentage of liver fat. The participants had no current or prior diagnosis of diabetes.

**Table 2.** Participant characteristics at the baseline

	Mean (sd)	Range
No. of participants (*n*)	10	
Age (year)	47⋅9 (15⋅7)	28⋅0–65⋅7
Body weight (kg)	67⋅9 (7⋅6)	55⋅3–82⋅1
BMI (kg/m^2^)^a^	25⋅5 (1⋅6)	23⋅4–28⋅1
Waist circumference^a^ (cm)	87⋅9 (6⋅4)	80⋅0–96⋅0
Hip circumference^a^ (cm)	99⋅1 (4⋅1)	92⋅0–104⋅5
Waist:hip ratio^a^	0⋅89 (0⋅06)	0⋅79–0⋅99
FINDRISC^b^	13⋅2 (3⋅2)	9⋅0–20⋅0
FBG (mM)	6⋅0 (0⋅4)	5⋅6–6⋅7
HbA_1c_^c^	39 (6⋅6)	33–54
Systolic blood pressure (mm Hg)	127 (16)	108–166
Diastolic blood pressure (mm Hg)	79 (13)	59–100
Fasting duration (h)	13⋅2 (1⋅2)	11⋅4–15⋅1
DXA total FM (kg)^+^ ^d^	24⋅0 (4⋅5)	15⋅3–31⋅1
DXA BF (%)^+^ ^d^	34⋅7 (6⋅7)	23⋅3–43⋅8
DXA total LM (kg)^+^ ^d^	43⋅3 (7⋅1)	36⋅4–58⋅4
DXA total FFM (kg)^+^ ^d^	45⋅6 (74⋅8)	38⋅5–61⋅6
DXA AbFM (kg)^+^ ^d^	2⋅1 (0⋅52)	1⋅5–3⋅1
MRI VAT:SAT ratio^e^	0⋅54 (0⋅19)	0⋅27–0⋅82
MRI pancreas fat (%)^e^	4⋅1 (1⋅6)	1⋅8–6⋅6
MRS liver fat (%)^e^	10⋅5 (13⋅7)	2⋅6–46⋅1

BMI, body mass index, with overweight cut points based on WHO recommendations^(28)^; FBG, fasting blood glucose, measured by fingerpick, glucose meter (CareSens®N, iSens, New Zealand); DXA, dual-energy X-ray absorptiometry; FM, fat mass; BF, body fat; LM, lean mass; FFM, fat-free mass (LM + BMC); AbFM, abdominal fat mass; MRI, magnetic resonance imaging; VAT, visceral adipose tissue; SAT, subcutaneous adipose tissue; MRS, magnetic resonance spectroscopy.

a Waist and hip circumference measured at screen visit.

b FINDRISC assessed at pre-screen based on reported risk measures, inclusion score ≥ 9; .

c Hb_A1c_ (*n* =9 participants) measured up to 28 d prior to the baseline.

d DXA (^+^*n* =8 participants) measured at screen visit.

e MRI and MRS measured at screen visit.

### Blood glucose response and incremental area under the glucose curve

**Fig. 2.**
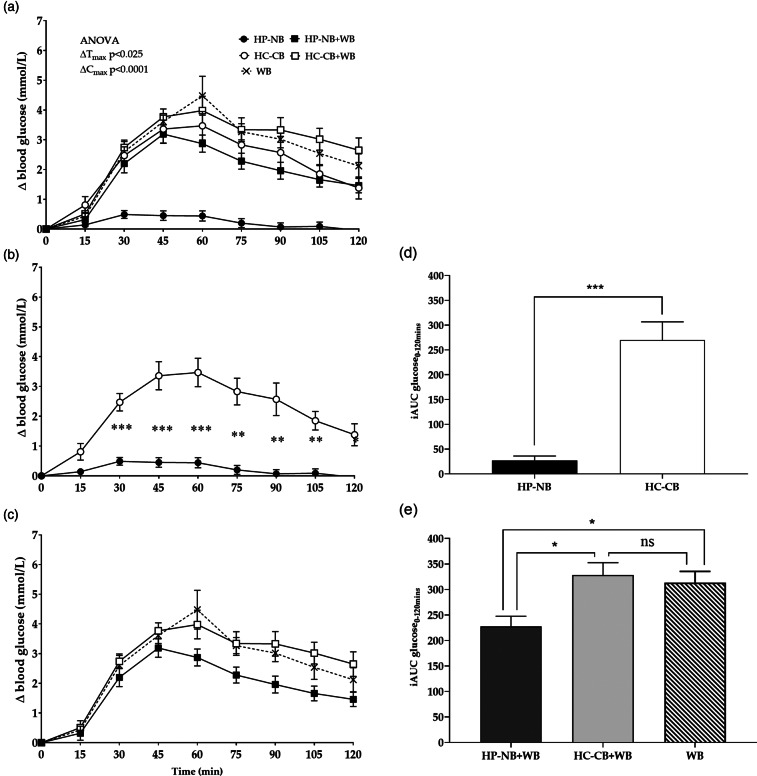
(a) Change in postprandial blood glucose concentration above the baseline (Δ blood glucose, mM; mean, sem) after consumption of the five test meals: HP-NB, higher-protein nut-based bar; HC-CB, higher-carbohydrate cereal-based bar; HP-NB + WB, higher-protein nut-based bar  +  white bread; HC-CB + WB, higher-carbohydrate cereal-based bar  +  white bread. ANOVA, Δ*C*_max_, treatment × time, *P* < 0⋅001, Δ*T*_max_, treatment × time, *P* < 0⋅025. (b) Iso-energetic test and control bars consumed alone: HP-NB, HC-CB. Tukey's *post-hoc* pairwise comparison, *P < 0⋅05. (c) Iso-energetic test and control bars co-ingested with WB: HP-NB + WB, HC-CB + WB, WB. Tukey's *post-hoc* pairwise comparison, HP-NB + WB *v.* WB, *P < 0⋅05. iAUC (iAUC_0–120min_) of glucose response over 2 h following consumption of test meals are shown as histograms; (d) ***P < 0⋅001 and (e) *P < 0⋅05.

Mean fasting FP blood glucose concentration at the baseline was not significantly different between the five meal treatments (ANOVA, *P* > 0⋅05) (Table 3). The time to maximum peak differed significantly between the five meals (Δ*T*_max_, ANOVA, *P* < 0⋅025, Fig. 2(a)), such that HP-NB reached the earliest postprandial glucose peak (mean Δ*T*_max_, sem: 39, 4⋅6 min), a consequence of the low-glycaemic response to this nut-based formulation. There was also a significant effect of meal treatment on postprandial blood glucose concentration for all measured parameters, including an increase in peak blood glucose concentration above the baseline (Δ*C*_max_, ANOVA, *P* < 0⋅0001; Fig. 2(a)) and iAUC blood glucose over 120 min (iAUC_0–120_, ANOVA, *P* < 0⋅0001; Fig. 2(d) and (e)). As hypothesised, Δ*C*_max_ was significantly lower following HP-NB (mean, sem: 0⋅8, 0⋅1 mmol/l) than the other four meal treatments, including the iso-energetic comparator HC-CB (3⋅8, 0⋅5 mmol/l, *P* < 0⋅01), HP-NB + WB (3⋅5, 0⋅3 mmol/l, *P* < 0⋅01), HC-CB + WB (4⋅5, 0⋅3 mM, *P* < 0⋅0001) and the WB-only treatment (4⋅8, 0⋅6 mmol/l, *P* < 0⋅01) (Table 3). When calculated over both 60 and 120 min, iAUC glucose was significantly lower for HP-NB than all other treatments (Table 3). When compared with HC-CB, iAUC_0–60_ and iAUC_0–120_ for HP-NB were approximately 6⋅5 (*P* < 0⋅001) and approximately 10 fold (*P* < 0⋅001, Fig. 2(d)) lower, respectively. Notably, the test product also ameliorated glycaemic response when co-ingested with WB, suppressing the high iAUC_0–120_ glucose of WB alone by almost one-third (−28 %, *P* < 0⋅05, Fig. 2(e)). This effect was not observed when HC-CB and WB were consumed together (*P* > 0⋅05) where there was no suppression of glycaemia relative to the high CHO WB meal treatment.

**Table 3.** Postprandial glycaemic response after consumption of the five meal treatments

Meal	Baseline glucose (mM)^1^	Δ glucose peak time Δ*T*_max_ (min)^2^	Δ glucose peak concentration Δ*C*_max_ (mM)^3^	iAUC_0–60min_ (mM× min)^4^	iAUC_0–120_ _min_ (mM × min)^4^
HP-NB	6⋅1 (0⋅1)	39⋅0 (4⋅6)^ab^	0⋅8 (0⋅1)^abcd^	19⋅1 (3⋅2)^abc^	27⋅2 (8⋅7)^abc^
HC-CB	5⋅9 (0⋅1)	54⋅0 (5⋅6)	3⋅8 (0⋅5)^a^	125⋅1 (14⋅3)^a^	269⋅7 (37⋅0)^a^
HP-NB + WB	6⋅1 (0⋅1)	48⋅0 (4⋅4)^b^	3⋅5 (0⋅3)^b^	108⋅2 (11⋅8)^b^	227⋅2 (20⋅4)^bc^
HC-CB + WB	5⋅9 (0⋅1)	67⋅5 (7⋅8)	4⋅5 (0⋅3)^c^	134⋅3 (8⋅4)^c^	327⋅9 (24⋅7)^c^
WB	5⋅8 (0⋅1)	58⋅5 (5⋅7)^a^	4⋅8 (0⋅6)^d^	132⋅3 (12⋅1)^b^	313⋅3 (22⋅2)^b^

Data are presented as mean, sem.

HP-NB, high-protein nut-based bar; HC-CB, high-carbohydrate cereal-based bar; WB, white bread; HP-NB + WB, high-protein nut-based bar + WB; HC-CB + WB, high-carbohydrate cereal-based bar + WB.

1 Baseline glucose (mM) presented as an average of FP blood glucose concentrations measured twice at −5 and −2 min before the start of test meal consumption. No significant difference between the five meal treatments at the baseline (*P* > 0⋅05).

2 Δ glucose peak time (min) is calculated as the time from the start of test meal consumption to the highest glucose concentration, using change (Δ) from baseline data.

3 Δ glucose peak concentration (mM) is calculated as maximum increase above pre-meal glucose levels, using change (Δ) from baseline data.

4 iAUC_0–60_ _min_ and iAUC_0–120_ _min_ for postprandial glucose response calculated as iAUC from 0 to 60 and 120 min using the trapezoidal method, including peaks above and beneath the baseline. Mean values in column with the same superscript are significantly different (*P* < 0⋅05).

**Fig. 3.**
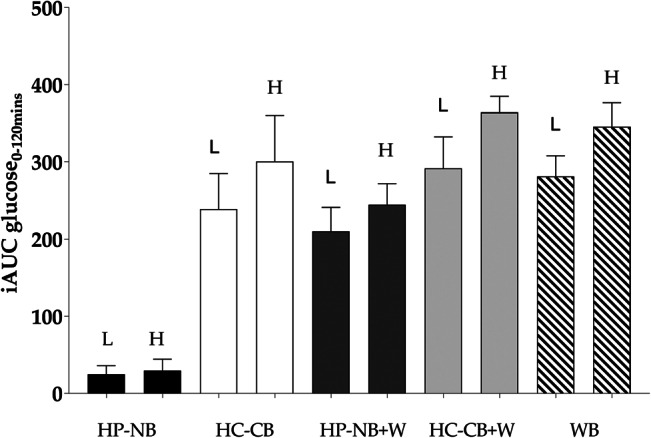
Mean (sem) iAUC (iAUC_0–120min_) of glucose response over 2 h following consumption of the five test meals in sub-cohorts of individuals with low (L, *n*=5) and high (H, *n*=5) pancreas and/or liver fat, assessed by MRI and MRS, respectively. High organ fat content significantly increased the glycaemic response when analysed across all meal treatments (ANOVA, *P* < 0⋅05).

Pancreas and liver fat varied greatly within the cohort. There was between 13 and 21 % greater suppression of iAUC_0–120_ glucose in the low pancreas and/or liver fat group when glycaemic response was compared between the high and low organ fat sub-groups for all participants across all five meal treatments (ANOVA, *P* < 0⋅05, Fig. 3), although the small sample size prevented statistical significance for the pair-wise meal treatment comparisons in this exploratory analysis. There was no difference in either BMI or DXA-assessed percentage of BF between these two sub-groups (25⋅1 *v.* 25⋅9 kg/m^2^; 35⋅6 *v.* 33⋅8 %, both *P* > 0⋅05), but there was evidence of a borderline higher baseline FBG in the high organ fat group (6⋅1 *v.* 5⋅8 mM, *P* = 0⋅09) prior to the meal challenge, which aligned with the exaggerated postprandial response. The suppression of glycaemia by HP-NB outlined earlier for the full cohort was still observed in the higher-risk high-organ-fat sub-cohort, including notably a 29 % decrease in the iAUC_0–120_ glucose of WB alone when co-ingested with the HP-NB test formulation.

### Palatability and appetite ratings

**Fig. 4.**
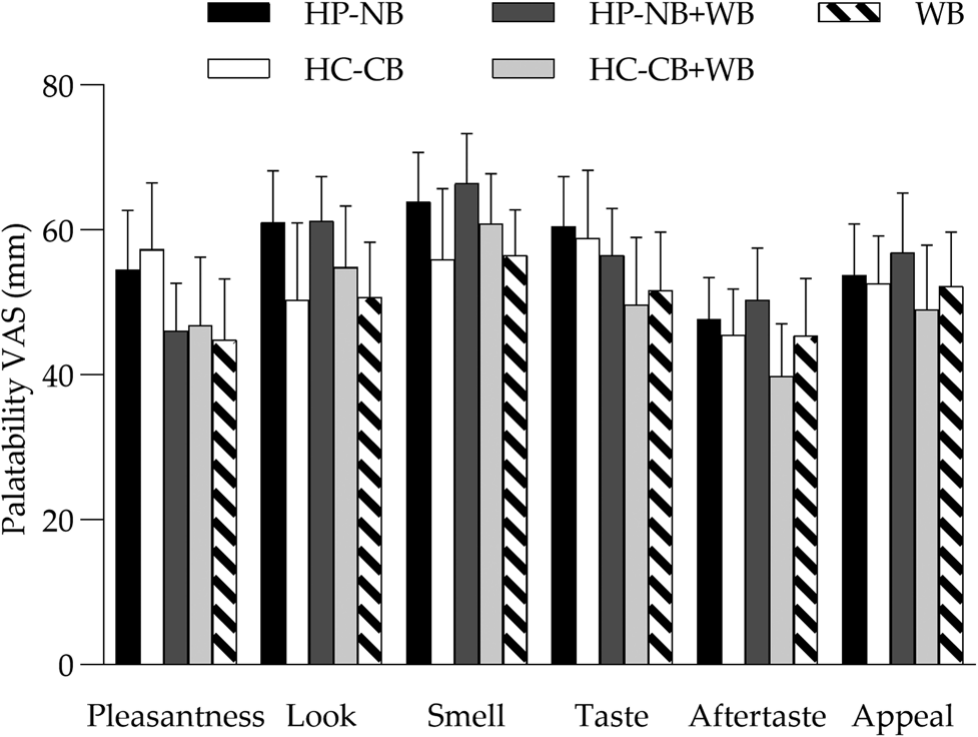
Mean (sem) VAS (mm) ratings of palatability (pleasantness, look, smell, taste, aftertaste, appeal) for the five test meals assessed immediately post consumption: HP-NB, higher-protein nut-based bar; HC-CB, higher-carbohydrate cereal-based bar; HP-NB + WB, higher-protein nut-based bar  +  white bread; HC-CB + WB, higher-carbohydrate cereal-based bar  +  white bread. No significant difference was observed between meals (*P* > 0⋅05).

**Fig. 5.**
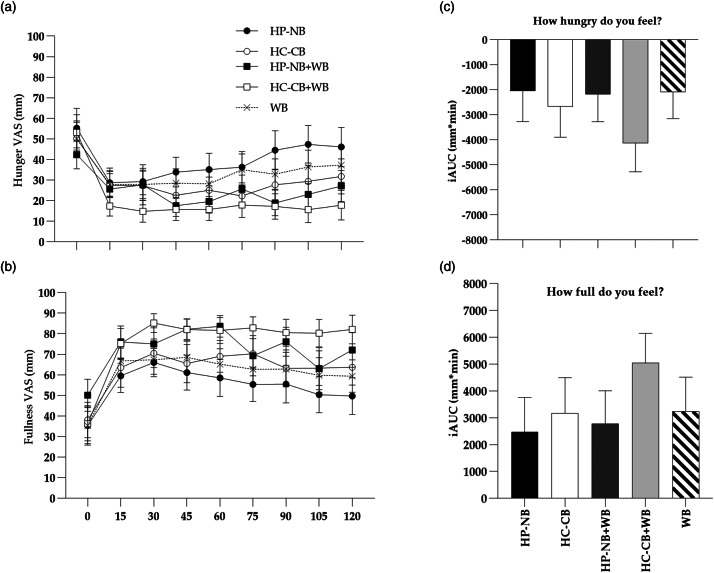
(a,b) Mean (sem) VAS ratings for hunger and fullness over 120 min for the five test meals: HP-NB, higher-protein nut-based bar; HC-CB, higher-carbohydrate cereal-based bar; HP-NB + WB, higher-protein nut-based bar + white bread; HC-CB + WB, higher-carbohydrate cereal-based bar + white bread. (c,d) iAUC (iAUC_0–120min_) of appetite response over 120 min following consumption of the test meals. There was no significant difference in pairwise comparisons of HP-NB test meal *v.* HC-CB comparator meal when consumed alone or during co-ingestion of WB (*P* > 0⋅05).

There was no significant difference in the palatability ratings of pleasantness, look, smell, taste, aftertaste and appeal between any of the five meals, when assessed on one occasion immediately following consumption of the meal (*P* > 0⋅05). The palatability ratings were high for both HP-NB and HC-CB (Fig. 4). There was no significant difference in VAS-appetite ratings between the HP-NB test and HC-CB comparator meals for hunger, fullness, satisfaction or TOF when analysed as iAUC_0–120_ using *post-hoc* pairwise comparisons (*P* > 0⋅05, Fig. 5). Hence, there was no evidence that HP-NB suppressed appetite relative to the iso-energetic HC-CB bar, either when consumed alone or when co-ingested with approximately 1 MJ WB. What must be noted here, however, is that this is a small sample study for VAS-appetite assessment.

## Discussion

In this study of overweight pre-diabetic Chinese adults, we showed that the incorporation of the recommended daily intake of mixed nuts (16–28 g)^(25,26,48)^ plus dried fruit into a higher protein, higher fibre, higher unsaturated fat snack formulation, in bar format, resulted in a significant suppression of glycaemic response, in strong contrast to an energy-matched high-cereal higher-CHO snack bar. While this was as expected, even in this dysglycaemic population, notably, the nut-based product also ameliorated the postprandial hyperglycaemia of a high CHO, high GI food item, namely, WB. Co-ingestion with the nut-based snack bar decreased the overall glycaemic impact by approximately 30 % in this high-risk group, even though the total CHO content ingested was increased.

Historically, incorporating nuts and dried fruits into the diet was discouraged based on their high fat and sugar content, respectively, and potentially adverse contribution to an energy-dense diet. In more recent years, however, both have become established as important food categories in a number of dietary regimes, including the Mediterranean diet^(25)^, with regular consumption of nuts, in particular, shown to improve a range of metabolic outcomes, including glycaemic control^(49)^. Several components may be responsible for the positive effects of our current test formulation containing peanuts and almonds, dried fruit and chicory oligofructose including higher-protein content, higher complex CHO and soluble fibre content, and higher unsaturated fat content, in addition to the higher polyphenol content relative to the comparator.

Plant-origin foods described using the generic term ‘nut’ have been defined as any edible, large, oily kernels within a shell and includes both tree (or ‘true’) nuts such as almonds, cashews, hazelnuts, macadamias, pistachios and walnuts and groundnuts such as peanuts. Peanuts grow as pods underground with an edible seed within the shell and are classified as legumes. Despite this major taxonomic difference from the tree nuts, peanuts also have an evidence base in support of their inclusion in the diet for improved metabolic health^(12,15,50–52)^. The nut portion of the snack bar in our present study was comprised of 50 % almonds and 50 % peanuts, with total of 29 g, meeting the recommended daily intake of nuts. Almonds have been the focus of a majority of nut trials. In healthy adults, almonds have been shown to decrease postprandial glycaemia and insulinaemia^(11,53,54)^, with some prior evidence of attenuation of the postprandial glycaemic response of WB^(21)^. In an early study, Josse *et al.*^(21)^ showed that co-ingestion of 30 g almonds with WB decreased peak glucose concentration by approximately 1⋅0 mM in comparison with WB alone, a comparable finding to our present study where glucose peak decreased by 1⋅3 mM following a similar intake of 29 g almonds and peanuts, despite the increase in total CHO. The authors concluded that this effect is likely due to a decrease in the rate of gastric emptying as a result of the addition of this high lipid and protein food source, in addition to the high fibre, polyphenol and phytic acid content of almonds^(21)^. Conversely, almond ingestion with a starchy buttered bagel and juice test meal failed to alter postprandial glucose peak in healthy normoglycaemic volunteers relative to the meal alone, but did suppress glycaemia in dysglycaemic T2D patients^(11)^. In adults with impaired glucose-tolerance, the consumption of almonds with a mixed meal also suppressed postprandial glycaemia^(54)^ and in a second trial in people with prediabetes, almonds given as a preload before a meal again decreased postprandial glycaemia^(55)^.

There is little data on glycaemic response to peanut consumption as yet available. Rodent studies have shown chronic peanut oil supplementation to decrease parameters including fasting glucose and Hb_A1c_^(15,50)^, with similar effects following peanut aqueous extract feeding studies^(56)^. Large observational studies such as the Nurse's Health Study have reported the contribution of peanuts to the positive effects of nuts on T2D prevention^(12,51)^, yet few RCTs have yet been conducted. In one of the few prior intervention studies, peanut consumption significantly suppressed postprandial glucose peak, again by approximately 1⋅0 mM, when consumed with a high-glycaemic bagel and juice meal in healthy participants^(57)^. Nut format and processing may also be of relevance in this high lipid-containing food. A high-dose intervention of ground and roasted peanuts within a breakfast meal resulted in a lowering of postprandial glycaemic response compared with whole raw peanuts^(58)^. A recent study by Lilly *et al.*^(59)^ also found that the supplementation of a high-GI meal with peanut butter attenuated the blood glucose spike and overall glycaemic response in healthy adults, while a study by Reis *et al.*^(17)^ observed that a high peanut butter bolus (42⋅5 g) with a CHO-rich breakfast decreased postprandial glycaemia relative to a nut-free breakfast-only control in obese women at an elevated risk for T2D. The addition of 42⋅5 g whole peanuts to a CHO-rich breakfast led to similar but non-significant effects. Also, the addition of the same amount of peanut butter suppressed the glycaemic response of the second-meal 4 h later. The greater bioaccessibility of the lipid component in the peanut butter was proposed to underpin this observation^(17)^. Furthermore, in a study of overweight and obese men, peanuts given as a high fat ‘thick shake’ were shown to decrease postprandial insulinaemia compared with a high-oleic peanut beverage^(60)^. High-oleic peanuts have previously been shown to increase diet-induced thermogenesis^(61)^, purportedly by increasing the gene expression of uncoupling proteins, and fat oxidation^(62)^ in overweight adults. Most recent is a study conducted in overweight Chinese adults with T2D, where a 12-week adherence to a peanut- or almond-supplemented low CHO diet for rapid weight loss was investigated^(52)^. Postprandial outcomes were also assessed, with both peanuts and almonds improving glycaemic parameters as expected under conditions of significant negative energy balance over 3 months, but with no reported differences between the legume and tree nut arms at any time point during the study, including at the baseline.

Also, recently, although with no direct relevance to our present trial in which the peanuts were served shelled and without skin, interestingly, peanut skin phenolic extract was shown to ameliorate postprandial glucose response in young healthy adults^(63)^. Previously, in a mechanistic study using Caco-2 cell lines, peanut skin procyanidins have been shown to inhibit α-amylase activity and decrease glucose transport *in vitro*^(64)^. Few studies have investigated the postprandial effect of peanuts when co-ingested with high GI food items. Johnson *et al.*, in 2005, reported significant postprandial amelioration when peanuts were co-ingested with a high (eighty-one units) but not a moderate (forty-eight units) GI meal in a cohort of eleven healthy adults^(57)^. Despite the major taxonomic differences between peanuts and the tree nuts and despite the relative paucity of data, evidence to date appears to support the inclusion of this unique legume with the tree nuts with respect to metabolic health improvements. More studies on this are required.

There is also a body of data that supports the inclusion of dried fruits in the diet of dysglycaemic individuals. Recently reviewed by Hernandez-Alonso *et al.*^(25)^, a majority of studies have been conducted with dried raisins and large dried grapes of characteristic black colour, with the weight of evidence showing beneficial postprandial effects on both insulin and glucose and on healthy, pre-diabetic and T2D cohorts. Commonly consumed dried fruits are typically high in both total CHO and fibre and have a low/moderate GI. Dehydrated apples included in our present trial as an ingredient in the nut and fruit-based formulation have a low GI of twenty-nine units and dehydrated blueberry also have a low GI ranging between forty and fifty-three units^(65)^.

The fibre content of the test formulation is likely to have played a major role in our present study observations, since dietary fibre is strongly associated with improved insulin sensitivity and consequent lower circulating insulin and glucose levels, including in patients with established T2D^(66)^. Soluble fibre is known to increase distension within the stomach and viscosity in the small intestine and decrease the rate of nutrient absorption^(67)^. Hence, CHO absorption and postprandial appearance of glucose in blood is significantly altered. A recent meta-analysis concluded that viscous fibre supplements should be recommended as part of T2D management, with significant improvement in Hb_A1c_, fasting glucose, fasting insulin and homeostatic model assessment of IR (HOMA-IR) relative to standard clinical care^(68)^. The inclusion of chicory oligofructose likely contributed to the glycaemic improvements observed with the HP-NB test bar, with a body of literature underpinning European Food Safety Authority (EFSA) substantiation of the amelioration of postprandial glycaemia when non-digestible CHO replaces at least 20 % by weight of available CHO in a high mono- or disaccharide product in the general population^(69)^. Non-digestible CHOs such as chicory oligofructose have been shown to be resistant to hydrolysis and absorption in the small intestine and so do not contribute to postprandial glycaemia. These prebiotics may, in turn, also contribute to chronic changes in the large bowel including fermentation to short-chain fatty acids and alteration of composition and function of the faecal microbiota, both of which, however, are hypothesised to alter fasting rather than postprandial glucose^(70)^. Nuts may also contribute as prebiotic food items with polymerised polyphenols and polysaccharides providing substrates for the gut microbiota^(71)^.

Also of note in our present trial was the exaggerated postprandial response to all meal treatments in the high-organ-fat sub-cohort. Albeit an exploratory analysis in a small sub-cohort not powered as primary or secondary outcome, there was up to a 20 % increase in area under the blood glucose curve over the 2 h test in individuals with MR-identified ectopic pancreas and/or liver fat infiltration. This is of physiological interest and worthy of further investigation in a larger well-powered cohort. Asian populations, including Chinese, are typified by a TOFI (‘thin on the outside fat on the inside’) phenotype\where, even at low BMI, greater VAT and/or ectopic fat may predominate. Hypothesised to be, at least in part, a consequence of limited and dysfunctional subcutaneous adipose depots^(72)^, even a small increase in body weight and adipose mass may result in this characteristic centralised fat deposition^(73)^ accompanied by worsening metabolic health, including dysregulated fasting and postprandial glycaemia and increased risk of T2D. Notably, in our cohort, there were significant correlations between both waist circumference and MRI-assessed VAT with the percentage of pancreas fat, while neither BMI nor DXA-assessed total percentage of BF were related to pancreas fat. Visceral adipose depots have long been associated with both worsened insulin sensitivity and IR^(74)^, and fatty liver and fatty pancreas^(75)^. Increased liver fat and high plasma triacylglycerol (TAG) concentrations expose pancreatic β-cells to excess circulating fatty acids and locally deposited tissue TAG, with the subsequent lipotoxicity inhibiting insulin secretion and promoting postprandial hyperglycaemia. The authors have proposed that a personal fat threshold for the β-cell may exist, which when exceeded may then worsen the risk of T2D^(76)^. In our cohort of ten adults with impaired fasting glucose, there was a borderline relationship between a higher percentage of pancreas fat and a higher FBG, calculated *post hoc* to have required fourteen participants in order to be identified as statistically significant.

There are some limitations to the data presented in this study. First, only blood glucose concentrations were measured. The interpretation of underpinning mechanisms could be expanded if other glycaemic endpoints were measured, in particular, the hormone insulin and C-peptide. This was also a small sample study in a single ethnicity. Strengths included the study methods, which were in accordance with the internationally recognised GI methodology of ‘International Standards Organization. Food products: determination of the glycaemic index (GI) and recommendation for food classification. 2010 (ISO 26642:2010(E)).’ The International Standard is based on a Joint FAO/WHO Expert Consultation, ‘Carbohydrates in human nutrition’ and designed to be used by research organisations.

## Conclusions

In this study of overweight, dysglycaemic Chinese adults, we have shown that a nut-based, higher-protein, fibre-enriched snack formulation, in bar format, suppresses postprandial glycaemic response, first as expected, when compared with a cereal-based higher-sugar bar, and second and most importantly, when co-ingested with a high GI food. Co-ingestion with the nut-based snack bar decreased the glycaemic impact of WB by almost 30 % despite a higher total CHO content of the meal. We have also shown preliminary data that support the hypothesis that the postprandial glycaemic response to a meal is dependent, at least in part, on fat deposition within key organs of pancreas and liver. This outcome requires replication in a larger cohort and other ethnicities.
